# TFF3 is a valuable predictive biomarker of endocrine response in metastatic breast cancer

**DOI:** 10.1530/ERC-15-0129

**Published:** 2015-06

**Authors:** Felicity E B May, Bruce R Westley

**Affiliations:** Department of Pathology, Faculty of Medical Sciences, Northern Institute for Cancer Research and Newcastle University Institute for Ageing, University of Newcastle-upon-Tyne, Framlington Place, Newcastle-upon-Tyne, NE2 4HH, UK

**Keywords:** breast cancer, trefoil factors, progesterone receptor, oestrogen receptor, antioestrogens

## Abstract

The stratification of breast cancer patients for endocrine therapies by oestrogen or progesterone receptor expression is effective but imperfect. The present study aims were to validate microarray studies that demonstrate TFF3 regulation by oestrogen and its association with oestrogen receptors in breast cancer, to evaluate TFF3 as a biomarker of endocrine response, and to investigate TFF3 function. Microarray data were validated by quantitative RT-PCR and northern and western transfer analyses. TFF3 was induced by oestrogen, and its induction was inhibited by antioestrogens, tamoxifen, 4-hydroxytamoxifen and fulvestrant in oestrogen-responsive breast cancer cells. The expression of TFF3 mRNA was associated with oestrogen receptor mRNA in breast tumours (Pearson's coefficient=0.762, *P*=0.000). Monoclonal antibodies raised against the TFF3 protein detected TFF3 by immunohistochemistry in oesophageal submucosal glands, intestinal goblet and neuroendocrine cells, Barrett's metaplasia and intestinal metaplasia. TFF3 protein expression was associated with oestrogen receptor, progesterone receptor and TFF1 expression in malignant breast cells. TFF3 is a specific and sensitive predictive biomarker of response to endocrine therapy, degree of response and duration of response in unstratified metastatic breast cancer patients (*P*=0.000, *P*=0.002 and *P*=0.002 respectively). Multivariate binary logistic regression analysis demonstrated that TFF3 is an independent biomarker of endocrine response and degree of response, and this was confirmed in a validation cohort. TFF3 stimulated migration and invasion of breast cancer cells. In conclusion, TFF3 expression is associated with response to endocrine therapy, and outperforms oestrogen receptor, progesterone receptor and TFF1 as an independent biomarker, possibly because it mediates the malign effects of oestrogen on invasion and metastasis.

## Introduction

Many of the 1.7 million women diagnosed each year with breast cancer benefit from endocrine therapy (May 2014). Introduced originally for the treatment of breast cancer patients who present or relapse following surgery with metastatic disease, endocrine therapy remains a mainstay of systemic intervention (Beatson 1896, Ariazi *et al*. 2006). Primary endocrine therapy is effective for patients with inoperable disease and for frail patients (Biganzoli *et al*. 2012). Adjuvant endocrine therapy for early breast cancer patients became widespread in the mid to late 1980s (Davies *et al*. 2013).

Two main categories of drugs target the dependence of malignant breast epithelial cells upon oestrogens. Aromatase inhibitors inhibit cytochrome P450 CYP-19, the enzyme that converts androgens to oestrogens, and thereby reduce circulating concentrations of tumour-promoting oestrogens (Lonning 1998, Santen *et al*. 2009). Letrozole and anastrozole, which were derived from aminoglutethimide, compete reversibly with haeme (Klein *et al*. 1995, Dowsett *et al*. 2000), whereas exemestane, which supplanted testolactone, competes irreversibly with the substrate (Geisler *et al*. 1998). Antioestrogens are competitive inhibitors of oestrogens for the oestrogen receptor, which is a ligand-dependent transcription factor with many target genes (May & Westley 1988, Wright *et al*. 2009). Tamoxifen and raloxifene have partial agonist activity (Powles 2013), whereas antioestrogens, such as fulvestrant, are pure antagonists (Howell *et al*. 2005, Johnston & Cheung 2010).

Around 50% of advanced breast cancer patients stratified to receive endocrine therapy based upon evaluation of oestrogen receptor status receive no benefit from the therapy, and some patients who are not offered endocrine therapy could benefit from the therapy (Chang *et al*. 2000, Allred 2010, Davies *et al*. 2011). The measurement of proteins encoded by genes whose expression is dependent upon an active oestrogen response should distinguish malignant cells that are oestrogen responsive and more sensitive to endocrine therapy. The detection of the progesterone receptor, which is induced by oestrogen, is indicative of a positive response (Horwitz *et al*. 1978, May *et al*. 1989, Osborne *et al*. 2005), and the detection of HER2, which is repressed by oestrogens, is indicative of a negative response to endocrine therapy (Allred 2010). *TFF1* is an oestrogen-responsive gene (Masiakowski *et al*. 1982, May & Westley 1986, 1988) that has utility as a marker of hormonal response in breast cancer patients, but it has not found widespread use (Foekens *et al*. 1990, Henry *et al*. 1990, 1991, Schwartz *et al*. 1991, Spyratos *et al*. 1994, Soubeyran *et al*. 1996).

The trefoil factor family includes the small secreted proteins TFF1, TFF2 and TFF3 (Thim & May 2005), which are expressed in and secreted from mucus secretory epithelia (Vestergaard *et al*. 2002, Samson *et al*. 2011). These proteins are involved in the protection and maintenance of healthy secretory epithelia via interactions with mucins and the stimulation of cell motility (Prest *et al*. 2002, Ruchaud-Sparagano *et al*. 2004, Thim & May 2005). TFF1 is expressed in normal and malignant breast epithelial cells (Rio *et al*. 1988, Henry *et al*. 1991, Piggott *et al*. 1991). TFF2 is expressed rarely in the breast (May *et al*. 2004). TFF3 mRNA is expressed in breast tumours and cell lines (May & Westley 1997, Poulsom *et al*. 1997), and TFF3 protein is expressed in normal breast epithelia and in a proportion of tumours (Ahmed *et al*. 2012). TFF3 protein expression is associated with lymph node involvement and local metastasis, and TFF3 mRNA expression is associated with cerebrospinal and osteo-metastasis (Smid *et al*. 2006, Kannan *et al*. 2010, Ahmed *et al*. 2012, May 2012, Magbanua *et al*. 2013).

Recent expression microarray analysis identified *TFF3* as one of relatively few genes whose expression is regulated consistently by oestrogen in oestrogen-responsive breast cancer cell lines (Wright *et al*. 2009). Furthermore, the *TFF3* gene is among the top genes whose expression correlates strongly with that of the oestrogen receptor in large cohorts of breast cancer patients (Gruvberger *et al*. 2001, West *et al*. 2001). The aims of the present study were to validate the microarray analyses, to determine the value of TFF3 as an independent predictive biomarker of response to endocrine therapy and to evaluate the response of breast cancer cells to TFF3.

## Materials and methods

### Cell culture

Breast cancer cells were obtained from the American Type Culture Collection (Manassas, VA, USA) or from their originators. Cell lines were verified as mycoplasma-free (MycoAlert Mycoplasma Detection kit; Lonza, Manchester, UK) and authenticated by short tandem repeat profiling at 16 loci by the LGC Standards (Middlesex, UK). MCF-7, T47-D, ZR-75, EFM-19, EFF-3, SK-Br-3, Hs 578T, BT-20, MDA-MB-231 and HBL-100 cells were cultured in DMEM containing 10% FCS (May & Westley 1988) and 1 μg/ml insulin (May & Westley 1988). Cells were discarded after the 30th passage. To measure the effects of oestrogen, cells were cultured for 5 days in phenol red-free DMEM containing 10% dextran-coated charcoal-treated new-born calf serum (DCC/CS) and 1 μg/ml insulin and then in this medium in the absence or presence of 10 nM oestradiol (E_2_) for 3 days (May & Westley 1986).

### Breast cancer cases

Ethical permission was obtained from the Joint Newcastle Health Hospitals and University of Newcastle-upon-Tyne Ethical Committee. Under the guidelines of the Human Tissue Act, specific consent was not requested retrospectively for archived tissue collected before 2006. For immunohistochemical analyses, 75 patients were identified who presented with symptomatic breast cancer between 1983 and 1986. Patients had had first-line surgery and received endocrine therapy as first-line systemic therapy either for existing advanced disease or upon relapse. All of the cases were invasive ductal carcinomas, apart from three invasive lobular carcinomas and two mucinous carcinomas. Endocrine therapy comprised of tamoxifen, aminoglutethimide or oophorectomy. For the validation cohort, a further 187 patients were identified who had had first-line surgery between 1992 and 1994.

Patients were followed-up clinically for 124 months or until death. Clinical data upon response to endocrine therapy was evaluated using standard criteria (Therasse *et al*. 2000). Response was categorised as complete response, partial response, static disease or progressive disease; the minimum criterion for response was disease that did not progress for at least 6 months.

### RNA and protein analysis

RNA was prepared by LiCl:urea precipitation and phenol:chloroform extraction as described previously (May & Westley 1986, Henry *et al*. 1990). RNA was analysed by northern transfer and RT-PCR as described previously (May & Westley 1988, Donaghue *et al*. 1999).

Recombinant human TFF3 was produced and purified (May *et al*. 2003), and the mature protein was analysed by ion exchange chromatography and gel filtration. Proteins were electrophoresed on nondenaturing and denaturing 12–32% polyacrylamide gels and were stained with Coomassie blue (May *et al*. 2003, Westley *et al*. 2005).

For western transfer analysis, proteins were transferred to 0.2 μm PVDF membrane, incubated with TFF3 antisera, conjugated secondary antibody and Pierce Supersignal reagent and exposed to X-ray film. For ELISA, wells were coated with 50 μl of 200 ng/ml peptide or 1–1000 ng/ml protein (Westley *et al*. 2005). Plates were washed and incubated sequentially with TFF3 monoclonal antibody hybridoma supernatant or mouse IgG, followed by the appropriate secondary antibody conjugated to alkaline phosphatase. The reaction was visualised with P-*N* phenyl phosphate (Piggott *et al*. 1991).

### Migration and invasion assays

For the migration assay, cells were plated into 16 mm-diameter underscored wells, washed twice with PBS and cultured in phenol red-free DMEM containing 10% DCC/CS for 24 h. The cell monolayer was ‘wounded’, washed twice with PBS and cultured for 2 h in 0.5 ml phenol red-free DMEM and 0.01% BSA. Cells were photographed and cultured in the absence and presence of TFF1 (Chadwick *et al*. 1997), TFF3 (May *et al*. 2003) or EGF (First Link, Wolverhampton, UK) for up to 7 h, at which point they were rephotographed. Cells were transfected with antisense RNA as described previously (de Blaquiere *et al*. 2009, Davison *et al*. 2011) and processed for the migration assay. Twenty measurements of each wound width were made, and the distance that the wound edge had moved was calculated.

For the invasion assay, phenol red-free DMEM and 0.01% BSA with and without recombinant TFF3 (May *et al*. 2003) were added to the bottom wells of a 48-well microchemotaxis chamber (Neuro Probe, Gaithersburg, MD, USA). A polyvinylpyrrolidone-coated polycarbonate membrane with 8 μm pores covered with collagen IV was laid over the wells. Cells were cultured in withdrawal medium for 2 days, trypsinised and allowed to recover for 1 h; then, 2.0×10^4^ cells in phenol red-free DMEM and 0.01% BSA were placed in each upper well. Cells were incubated at 37 °C, stained and counted (Prest *et al*. 2002).

### Antibody production

Rabbits were immunised with a 15 amino acid residue TFF3 peptide conjugated to keyhole limpet haemocyanin. BalbC mice were immunised with recombinant TFF3, mixed with alum or titre max gold as adjuvant and boosted at 2-week intervals. The titre of anti-TFF3 antibodies during the immunisation was monitored by ELISA. Spleen cells from immunised mice were fused with NS-1 cells and hybridomas that produce anti-TFF3 antibodies identified by ELISA. Hybridomas were cloned by limiting dilution at least thrice.

### Immunohistochemistry

Whole sections of formalin-fixed, paraffin-embedded tissue were deparaffinised, rehydrated and incubated in 0.5% hydrogen peroxide for 10 min and trypsin for 10 min or 10 mM citrate buffer (pH 6.0) in a pressure cooker for 1 min. Endogenous biotin was blocked with the Dako Cytomation. Sections were incubated with monoclonal antibodies against TFF3 protein, TFF1 protein (Westley *et al*. 2005), oestrogen receptor (NCL-L-ER-6F11; Leica Biosystems, Newcastle Upon Tyne, UK) or progesterone receptor (NCL-L-PGR-312; Leica Biosystems), secondary antibody and avidin-biotin immunoperoxidase complex (Vector Laboratories, Peterborough, Cambridgeshire, UK), developed with diaminobenzidine and counterstained with haematoxylin.

### Statistical analysis

Differences between expression levels and differences between stimulation of migration or invasion were tested by ANOVA. Results were considered to be statistically significant if *P*<0.05. For the immunohistochemical work, a histoscore that combined the percentage of immuno-reactive malignant cells with the intensity of the immuno-reaction was generated (Crosier *et al*. 2001, Ahmed *et al*. 2012). The statistical significance of differences observed was analysed with SPSS Software version 19.0 (SPSS, Inc.) by Kruskal–Wallis or Mann–Whitney *U* tests, and the statistical significance of correlations was analysed by Pearson's correlation or Spearman's *ρ* correlation. The predictive value of the biomarkers as continuous variables was tested by linear regression and binary logistic regression analyses, and their sensitivity and specificity were tested in a binary classification test by *χ*^2^ analysis. Multivariate analyses were adjusted for potential confounding variables. Parameters were scaled, and the predictive ability of the continuous variables was evaluated by multiple binary logistic regression with the Wald statistic and by multiple linear regression analysis refined by stepwise selection.

## Results

### TFF3 regulation and association with oestrogen receptor

**Figure 1:**
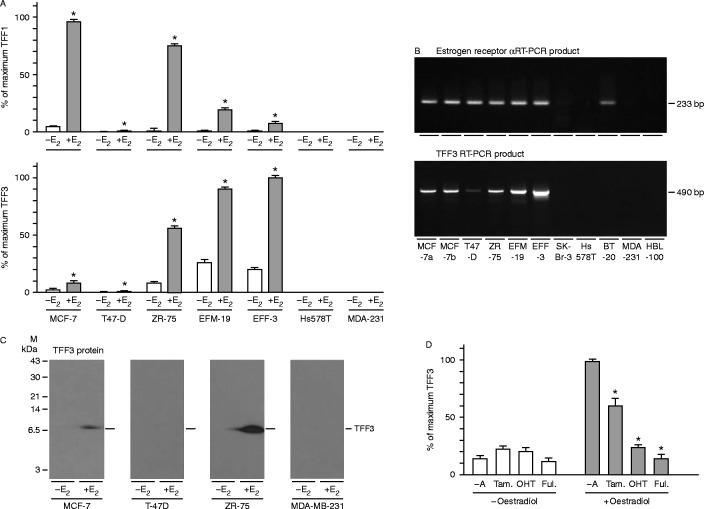
Oestrogen dependence of TFF3 expression in breast cancer cells. MCF-7, T47-D, ZR-75, EFM-19, EFF-3, SK-Br-3, Hs 578T, BT-20, MDA-MB-231 (MDA-231) and HBL-100 cells were withdrawn from hormones present in routine culture medium and incubated in the absence (−E_2_) and presence of 10 nM oestradiol (+E_2_) (A and C), and in the absence (−A) and presence of tamoxifen (Tam), 4-hydroxytamoxifen (OHT) or fulvestrant (Ful; D), or they were grown in maintenance medium (B). RNA was prepared and amplified by quantitative real-time PCR (A). RNA was prepared from cells grown in maintenance medium, amplified with primer pairs for the oestrogen receptor (490 bp) and TFF3 (233 bp) mRNAs, and the PCR products were separated by gel electrophoresis on a 3% agarose gel and stained with ethidium bromide (B). MCF-7, T-47D, ZR-75 and MDA-MB-231 cells were gown in the absence (−E_2_) and presence of 10 nM oestradiol (+E_2_) and lysed, and protein extracts were prepared. The proteins were transferred to PVDF membrane, and TFF3 was measured by western transfer analysis (C). The positions of the molecular mass markers are shown on the left, and those of TFF3 are shown on the right of the panels. The means±s.e.m. of at least three replicates are shown (A and D; Δ*P*<0.05).

Expression microarray analysis (Wright *et al*. 2009), which demonstrated consistent regulation of TFF1 and TFF3 but not TFF2 in three oestrogen-responsive breast cancer cell lines, was validated by real-time RT-PCR (Fig. 1). TFF1 and TFF3 mRNAs were induced by oestrogen in five oestrogen-responsive but not in two oestrogen-nonresponsive breast cancer cell lines. Because the importance of the regulation of TFF3 by oestrogen in breast cancer is unknown, unlike that of TFF1, it was studied further. Oestrogen-receptor and TFF3 mRNAs were co-expressed in six of 11 cell lines, and neither was expressed in five of the cell lines. BT20 cells, which are known to express aberrant oestrogen receptor but to be unresponsive to oestrogens, did not express TFF3 mRNA. TFF3 protein was not detected in the oestrogen-unresponsive MDA-MB-231 cells or in T-47D cells, which expressed low levels of TFF3 mRNA. More TFF3 protein was detected in ZR-75 cells and MCF-7 cells that had been grown in the presence of oestrogen than in those that had been grown in its absence. TFF3 expression was not induced by tamoxifen, 4-hydroxytamoxifen or fulvestrant, but all three antioestrogens inhibited the induction of TFF3 by oestrogen.

**Figure 2:**
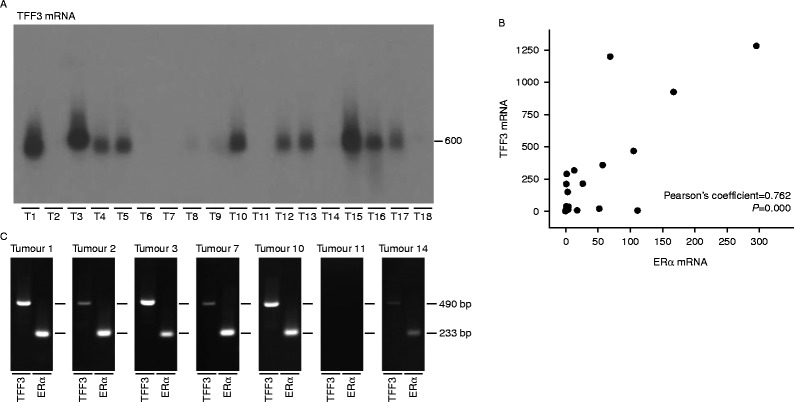
Association between oestrogen receptor and TFF3 mRNAs in primary breast tumours. RNA was prepared from primary breast tumours. Ten microgram of breast tumour RNA were separated by gel electrophoresis and transferred to Hybond-N membranes, and TFF3 mRNA (600 nucleotides) was detected by hybridisation (A). The association between TFF3 mRNA and oestrogen receptor mRNA expression in each primary tumour is shown (B). The Pearson's coefficient and *P* value are indicated on the panel. RNA was reverse transcribed and amplified by PCR with the primer pairs for the oestrogen receptor (233 bp) and TFF3 (490 bp) mRNAs. The PCR products were separated by gel electrophoresis on a 3% agarose gel and stained with ethidium bromide (C).

The expression of *TFF3* was measured next by northern transfer and RT-PCR analysis in a panel of breast tumours. The amount of TFF3 mRNA varied enormously; there were high levels in ten, moderate levels in two, low levels in four and none in two tumours (Fig. 2). There was a strong positive association between oestrogen receptor and TFF3 mRNA expression (Pearson's coefficient=0.762, *P*=0.000). The results from the RT-PCR analysis agreed well with those from the northern analysis. Despite the strong correlation between oestrogen receptor and TFF3 mRNA expression, the association was not absolute. For instance, there was high expression of oestrogen receptor but not TFF3 mRNA in tumours 2 and 7 and high expression of TFF3 but not oestrogen receptor mRNA in tumour 3.

### The production and evaluation of antibodies against human TFF3 protein

The data indicated that TFF3 merited investigation as an oestrogen-responsive biomarker in breast cancer patients. Antibodies were generated for immunohistochemical analysis of TFF3 expression. TFF3 protein was chosen as the immunogen as opposed to synthetic TFF3 peptides because the resultant antibodies should have superior sensitivity and specificity (Westley *et al*. 2005). Recombinant human TFF3 protein was produced and purified to homogeneity as evaluated by ion exchange chromatography and electrophoresis under nondenaturing and denaturing conditions. It had the predicted molecular mass of 6.6 kDa (May *et al*. 2003).

**Figure 3:**
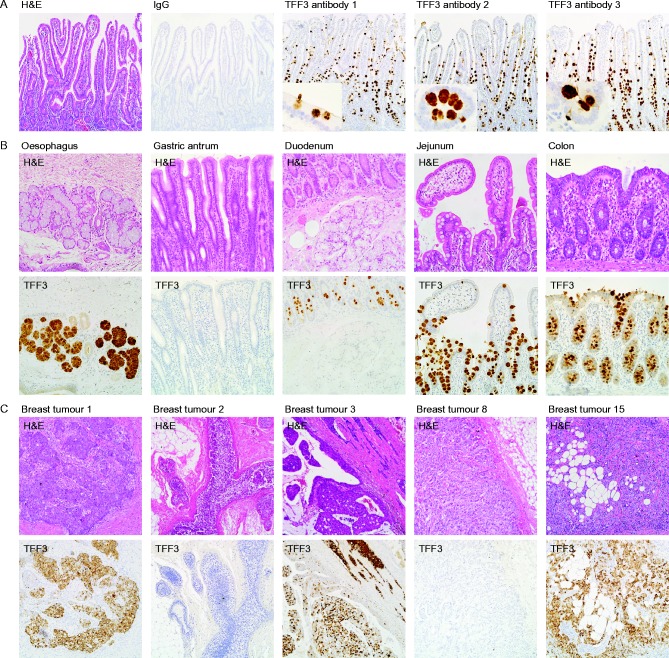
Immunohistochemical validation of the antibodies against human TFF3 protein. Sections of human ileum (A), oesophagus, gastric antrum, duodenum, jejunum, and colon (B) and of primary breast tumours (C) were stained with haematoxylin and eosin or processed for immunohistochemistry with antibodies against mouse IgG or TFF3 as indicated. The original magnifications for the photomicrographs were ×100 (A and C), ×200 (B) and ×400 for the inserts in (A). A full colour version of this figure is available at http://dx.doi.org/10.1530/ERC-15-0129.

Hybridomas were generated from mice that had been immunised with purified TFF3, and the sensitivity and specificity of the monoclonal antibodies were evaluated by ELISA and immunohistochemistry. The antibodies detected less than 1 ng of human TFF3 and did not react with TFF1 or TFF2 or with other growth factors (data not shown). The antibodies reacted strongly with TFF3 in the mucus secretory granules of goblet cells in human ileum (Fig. 3). Equally strong immuno-reaction was detected in the secretory granules of neuroendocrine cells. Less immuno-reaction was visible in the cytoplasm of goblet cells, and none was observed in the cytoplasm of absorptive intestinal cells. There was no immuno-reaction with stromal or endothelial cells. The antibodies detected TFF3 in oesophageal submucosal glands but not in the surface epithelium or in normal gastric mucosa. TFF3 was absent from Brunner's glands, but it was detected in all goblet cells of the duodenum and throughout the intestines.

Strong immuno-reaction was detected in the cytoplasm of malignant breast epithelial cells of tumours that expressed high concentrations of TFF3 mRNA (tumours 1, 3 and 15; Fig. 3). A much weaker reaction was detected in tumours with low TFF3 mRNA concentrations (tumours 2 and 8), and there was no reaction in tumours in which TFF3 mRNA was undetectable. These results demonstrate excellent congruence between TFF3 mRNA and protein expression and suggest that the anti-TFF3 protein antibodies are specific and sensitive.

### The expression of TFF3 in primary breast tumours and its association with oestrogen receptor, progesterone receptor and TFF1 expression

To evaluate the potential of TFF3 as a predictive biomarker of response to endocrine therapy, we identified a cohort of advanced breast cancer patients who had received endocrine therapy without stratification. Serial sections of the primary breast tumours were analysed by immunohistochemistry.

**Figure 4:**
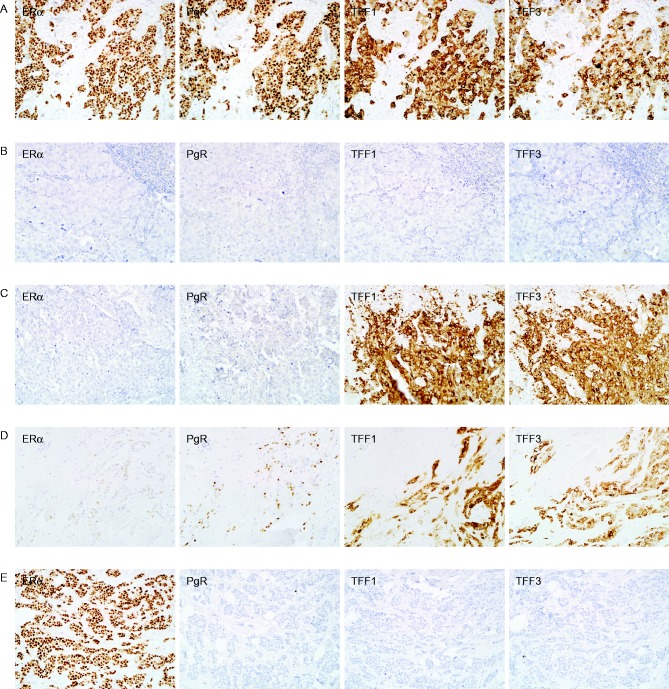
The expression of oestrogen receptor, progesterone receptor, TFF1 and TFF3 in breast cancers. Sections of primary breast tumours were processed for immunohistochemistry to measure oestrogen receptor (ERα), progesterone receptor (PgR), TFF1 and TFF3. Sections are from tumours in which all four biomarkers were detected (A), in which none of the biomarkers were detected (B), in which one or more of the oestrogen-responsive biomarkers was detected in the absence (C) or presence (D) of low oestrogen-receptor expression and in which the oestrogen receptor is expressed with low associated expression of the three responsive genes (D). The original magnifications for the photomicrographs were ×200. A full colour version of this figure is available at http://dx.doi.org/10.1530/ERC-15-0129.

TFF3 and TFF1 were detected in the cytoplasm, whereas oestrogen and progesterone receptors were detected in the nuclei of normal and *in situ *and invasive malignant breast epithelial cells. The expression of all four biomarkers was detected in some tumours, whereas none of the biomarkers were expressed in other tumours (Fig. 4). In some tumours, one or more of the oestrogen-responsive proteins was expressed in the absence of detectable oestrogen receptor expression or in the presence of low oestrogen receptor expression, and in a few tumours, strong oestrogen receptor expression was present with no or low expression of the three oestrogen-responsive proteins.

**Figure 5:**
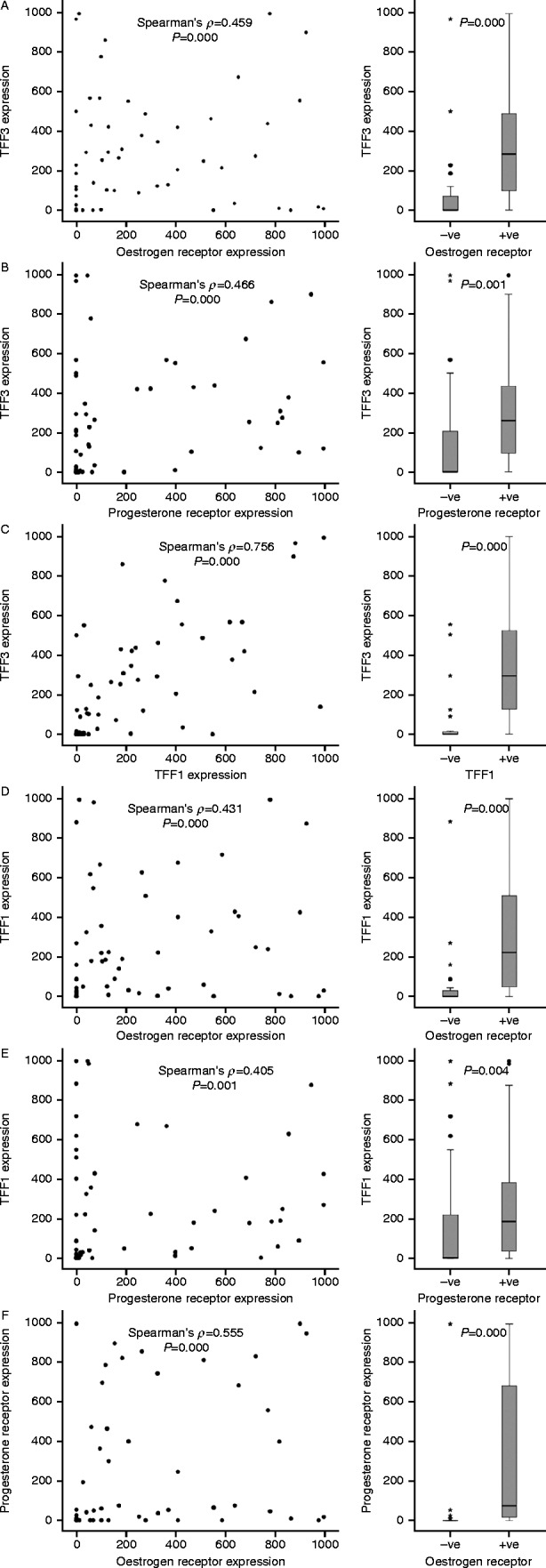
Associations between the expression of TFF3 and the other three biomarkers. The TFF3 histoscore in individual tumours was compared with the expression of oestrogen receptor (A), progesterone receptor (B) and TFF1 (C). The associations between TFF1 and oestrogen receptor (D) and progesterone receptor (E), and those between the oestrogen and progesterone receptors (F), are shown also. For each paired association, the Spearman's *ρ* and *P* values are indicated on the panels. For the panels on the right, the comparators were dichotomised by Allred of >3.0 (A, B, D, E and F) or a histoscore of >35 (C). The horizontal bars represent the median values, the boxes indicate the range of the second and third quartiles of the data and the whiskers represent the range of all the data. The circles represent outliers of more than 1.5 times, and the stars represent extreme values of more than 3.0 times farther from the third quartile than the difference between the third quartile and the median.

TFF3 was detected in 71% of the breast tumours. There was a strong positive correlation between TFF3 and oestrogen receptor expression (Spearman's *ρ*=0.459, *P*=0.000), which is consistent with TFF3 expression being under the control of oestrogen (Fig. 5). TFF3 expression was higher in oestrogen receptor-positive tumours (Allred ≥3.0) (Allred 2010) than it was in oestrogen receptor-negative tumours (Mann–Whitney *U* test, *P*=0.000).

There was a strong positive correlation between the expression of TFF3 and the progesterone receptor (Spearman's *ρ*=0.466, *P*=0.000) and between TFF3 and TFF1 (Spearman's *ρ*=0.756, *P*=0.000), which is consistent with the co-regulation by oestrogen of TFF3 and the other two oestrogen-responsive biomarkers (Fig. 5). TFF3 was higher in progesterone receptor-positive tumours as compared to progesterone receptor-negative tumours (Mann–Whitney *U* test, *P*=0.001) and in TFF1-positive tumours as compared to TFF1-negative tumours (Mann–Whitney *U* test, *P*=0.000).

We investigated if the expression of TFF1 was associated, like TFF3, with that of the oestrogen or progesterone receptor. There was a correlation between TFF1 and oestrogen receptor expression (Spearman's *ρ*=0.431, *P*=0.000) and progesterone receptor expression (Spearman's *ρ*=0.405, *P*=0.001). TFF1 expression was higher in tumours that express oestrogen receptors (Mann–Whitney *U* test, *P*=0.000) and progesterone receptors (Mann–Whitney *U* test, *P*=0.004). The associations between TFF1 and the oestrogen and progesterone receptors were slightly weaker than those of TFF3. Lastly, the relationship between the expression of the progesterone and oestrogen receptors was tested. Expression of the two receptors was associated (Spearman's *ρ*=0.555, *P*=0.000), and expression of progesterone receptor was higher in tumours that express oestrogen receptor than it was in those that do not (Mann–Whitney *U* test, *P*=0.000). Thus, the expression of all four biomarkers is correlated, but there is sufficient divergence to indicate that they might provide independent predictive information.

### Association between TFF3 and response to endocrine therapy

**Figure 6:**
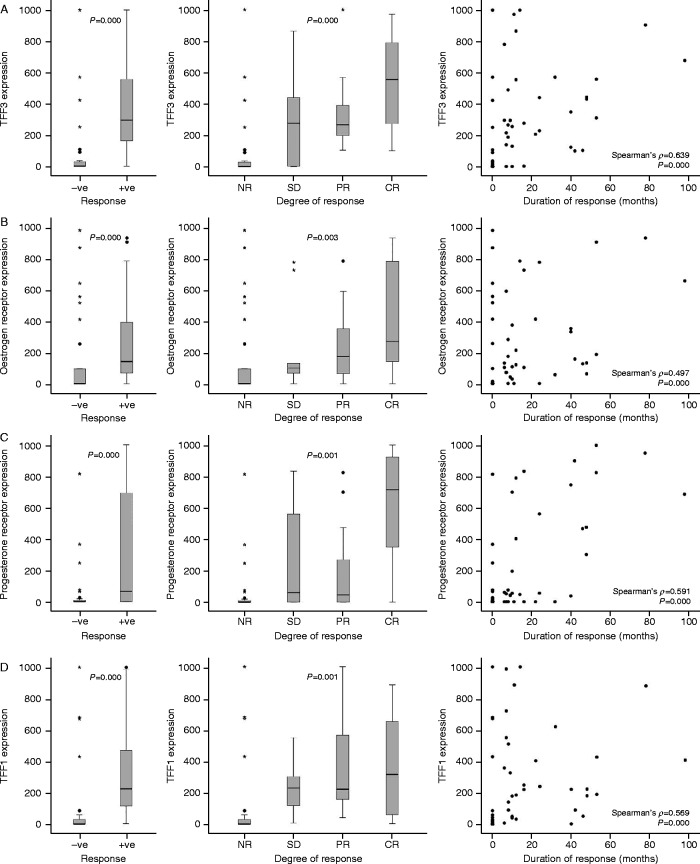
The association of TFF3, TFF1, progesterone receptor and oestrogen receptor expression with response to hormonal therapy. TFF3 expression (A) was compared in malignant cells in tumours from patients without (−ve) and with (+ve) a response to endocrine therapy as well as from patients whose disease progressed (NR), stabilized (SD) or demonstrated a partial (PR) or complete response (CR) to endocrine therapy. Similar analyses are shown for TFF1 (B), progesterone receptor (C) and oestrogen receptor (D). The horizontal bars represent the median values, the boxes indicate the range of the second and third quartiles of the data and the whiskers represent the range of all the data. The circles represent outliers of more than 1.5 times, and the stars represent extreme values of more than 3.0 times farther from the third quartile than the difference between the third quartile and the median. The associations between each biomarker and the duration of response are shown. For each paired association, the Spearman's *ρ* and *P* values are indicated on the panels.

Almost half of the patients responded to endocrine therapy. Of the tumours illustrated in Fig. 4, a complete response was obtained for two patients (A and C), one had static disease (D), and there was no response for two (B and E). TFF3 expression was higher in the tumours of women who responded to endocrine therapy than in those of women who received no benefit (*P*=0.000, Mann–Whitney *U* test, *Z*=−4.940) (Fig. 6). TFF3 expression was associated with the degree of response (Kruskal–Wallis II test *P*=0.000); patients with stable disease or partial response had TFF3 expression that was intermediate between those that did not respond and those with a complete response. The median value of TFF3 was considerably higher (500-fold) in patients with a complete response than it was in patients with progressive disease. TFF3 expression was correlated also with the duration of response to endocrine therapy (Spearman's *ρ*=0.639, *P*=0.000).

Oestrogen receptor, progesterone receptor and TFF1 expression were associated also with response to hormone therapy, although to different extents (Fig. 6). Expression was significantly higher in tumours from women who responded to hormone therapy than in tumours from those who did not (oestrogen receptor: *P*=0.000, Mann–Whitney *U* test, *Z*=−3.555; progesterone receptor: *P*=0.000, Mann–Whitney *U* test, *Z*=−3.601; and TFF1: *P*=0.000, Mann–Whitney *U* test, *Z*=−4.758), but the magnitude of the differences between those who responded and those who did not was smaller than that for TFF3 (Kruskal–Wallis II test *P*=0.003, *P*=0.001 and *P*=0.001 respectively). Levels of expression were related to the degree of response, and the highest level of expression for all of the biomarkers was in tumours of patients with complete response. The expression of oestrogen receptor, progesterone receptor and TFF1 was correlated with the duration of response, but the correlations were weaker than they were for TFF3.

### TFF3 as a biomarker of response to endocrine therapy

We evaluated the potential of TFF3 to be an effective predictive biomarker of endocrine response by univariate logistic regression analysis. TFF3 was a statistically significant predictor of hormonal response (*P*=0.000). Of the biomarkers considered, TFF3 was the most effective univariate predictor, followed by progesterone receptor (*P*=0.001), TFF1 (*P*=0.002) and oestrogen receptor (*P*=0.082). Univariate analyses by binary logistic regression analyses for the four biomarkers gave similar results: TFF3, progesterone receptor, TFF1 and oestrogen receptor (*P*=0.001, *P*=0.002, *P*=0.002 and *P*=0.079 respectively).

The specificity and sensitivity of the biomarkers was investigated with a binomial classification test. TFF3 and TFF1 expression was dichotomised at a histoscore of >35, and oestrogen and progesterone receptors were dichotomised at an Allred score of ≥3. TFF3 was an effective predictor, with a sensitivity of 91% and a specificity of 79% (*χ*^2^; *P*=0.000), which indicates that TFF3 has utility for determining both which patients will benefit from endocrine therapy and which patients will not. TFF1 was effective with a sensitivity of 90% and a specificity of 73% (*χ*^2^; *P*=0.000). Oestrogen and progesterone receptors gave sensitivities of 91 and 69% and specificities of 69 and 72% respectively (*χ*^2^; *P*=0.000). Oestrogen receptor and TFF3 were the most sensitive biomarkers, and TFF3 was the most specific biomarker.

### Multivariate analyses

The relative importance of TFF3 as a predictive biomarker of endocrine response was evaluated by multivariate binary logistic regression analysis. Amongst the clinico-pathological features and molecular biomarkers considered, TFF3 had the greatest ability to predict the endocrine response (*P*=0.000), followed closely by progesterone receptor (*P*=0.002). None of the other factors considered, including age, tumour size, tumour type, oestrogen receptor or TFF1, made a significant contribution to the prediction. The regression equation was:



TFF3 expression predicted the degree of response as a univariate biomarker (*P*=0.002). The ability of the different variables to predict the degree of response was tested by multiple linear regression analysis. TFF3 (*P*=0.000) and progesterone receptor (*P*=0.000) were retained in the model as effective independent predictors of the degree of response to endocrine therapy.

TFF3 expression predicted also response duration as a univariate biomarker (*P*=0.002). After evaluation of the ability of the different variables to predict response duration by multiple linear regression analysis, progesterone receptor (*P*=0.005) and TFF1 (*P*=0.034) were retained as independent predictors of the length of response to endocrine therapy.

The predictive values of TFF3, TFF1, progesterone receptor and oestrogen receptor were investigated in a separate cohort of patients with metastatic breast cancer. Univariate binary logistic regression analysis demonstrated that the biomarkers are statistically significant predictors of hormonal response. Multivariate binary logistic analysis showed that TFF3 was the most effective predictive biomarker (*P*=0.000). The predictive value of the progesterone receptor was slightly higher (*P*=0.001) in the validation than it was in the discovery cohort. Thus, the analysis of a second group of patients corroborates the potential of TFF3 to be a predictive biomarker of endocrine sensitivity.

### Effects of TFF3 on migration and invasion

**Figure 7:**
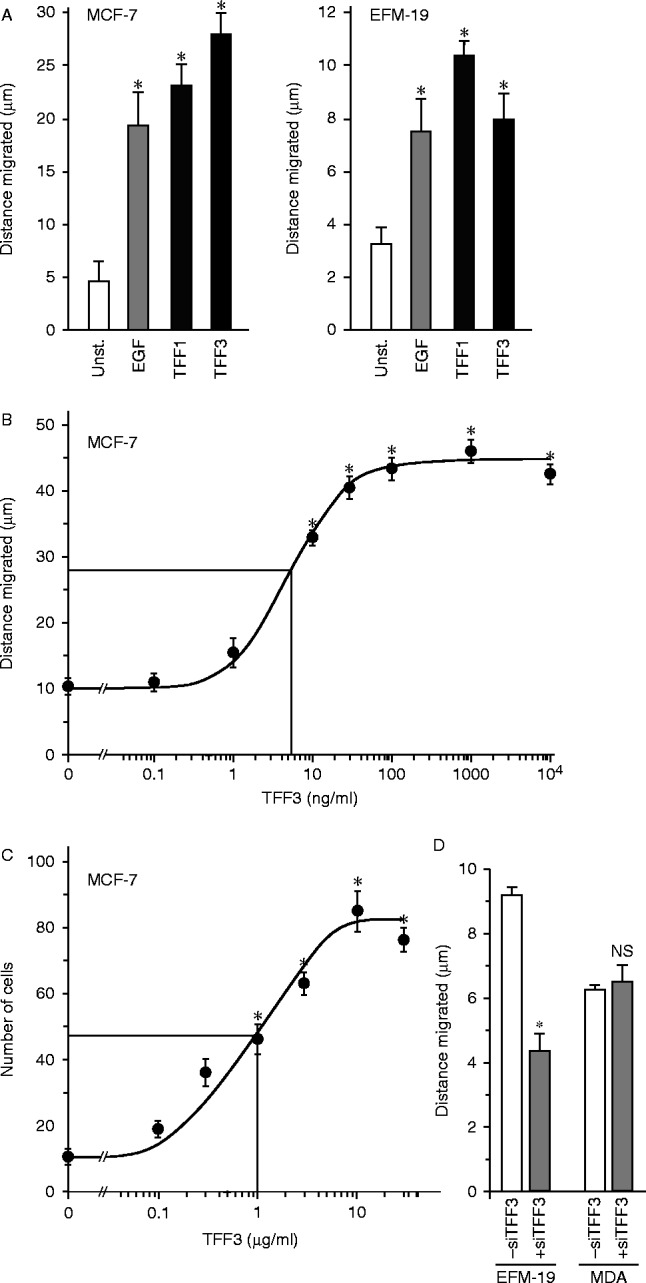
The effects of TFF3 on breast cancer cell motility and invasion. Monolayers of cells were ‘wounded’ with a pipette tip (A, B and D), and incubated in the absence (unst.) or presence of 10 ng/ml EGF or 5 μg/ml TFF1 or TFF3 for 5 h (A) or the indicated concentration of TFF3 for 7 h (B) or transfected with antisense TFF3 RNA and incubated for 5 h (D). The distance that the wounded edges moved was measured, and the mean distances±s.e.m. of at least four measurements is shown. For the invasion assay, 2.0×10^4^ cells were placed in the upper wells of a microchemotaxis chamber in medium containing 0.01% BSA. Cells were left to migrate on and through 8 μm pore polyvinylpyrrolidome membranes covered with collagen towards the lower wells, which contained the same medium but different concentrations of TFF3. Cells were counted in five high-power fields from triplicate wells, and the means±s.e.m. are shown (C). *Cell movement is statistically significantly higher than for unstimulated cells or lower than for cells transfected with TFF3 siRNA (ANOVA *P*<0.05).

Invasive and metastatic cells must be able to move through the stroma and travel to distant sites. We investigated if the predictive ability of TFF3 might be connected with an ability to mediate malign effects of oestrogen on breast cancer cell migration and invasion. TFF3 stimulated the migration of breast cancer cells in an *in vitro* wounding assay (Fig. 7A). The magnitude of migration stimulated by TFF3 was similar to the effects of TFF1 and EGF. The motogenic effect of TFF3 was concentration dependent (Fig. 7B). TFF3 stimulated also the invasion of breast cancer cells through collagen IV in a modified Boyden chamber assay (Fig. 7C). The motogenic effects of TFF3 were abrogated by antisense RNA in cells that express TFF3 but not in cells that do not express TFF3.

## Discussion

The present study validated expression microarray analyses that identified consistent oestrogen regulation of TFF3 mRNA in breast cancer cells and an association between oestrogen receptor mRNA and TFF3 mRNA expression in primary breast tumours (Gruvberger *et al*. 2001, West *et al*. 2001). Our demonstration that TFF3 protein is regulated by oestrogen and inhibited by antioestrogens in oestrogen-responsive breast cancer cells supports the potential of TFF3 protein as a biomarker of oestrogen dependence. That TFF3 stimulates migration and the invasion of breast cancer cells suggests that removal of these effects during anti-endocrine therapy would be beneficial to patients in whose tumour cells TFF3 is expressed under the control of oestrogens.

The antibodies produced against the correctly folded TFF3 protein (Muskett *et al*. 2003) detect human TFF3 with specificity and sensitivity. TFF3 is a secreted protein and was detected exclusively in the cytoplasm or mucus-secretory vesicles; there was no evidence of a nuclear immuno-reaction, as has been reported with some commercially available antibodies. TFF3 expression was detected in the submucosal glands of the oesophagus and in neuroendocrine and goblet cells throughout the small and large intestines. TFF3 was expressed in Barrett's metaplasia of the oesophagus and in intestinal metaplasia of the stomach. There was a good correlation between breast tumour TFF3 mRNA and protein expression.

TFF3 protein was expressed in the majority of the breast tumours analysed. A strength of the present study is that whole sections of tumour tissue, rather than small cores of tissue, were analysed. In tumours with stronger TFF3 expression, expression was distributed relatively evenly throughout the tumour, whereas in other tumours, expression was strong in some areas and undetectable in others. The positive association between oestrogen receptor and TFF3 expression indicates that TFF3 expression is oestrogen dependent in a majority of breast tumours. The strong association between the expression of TFF3 and the other two oestrogen responsive proteins supports this contention. The close relationship between TFF3 and TFF1 may reflect the co-ordinated expression of the two genes from the same genomic locus; they are separated by 55 kb. The expression of TFF3 in the absence of oestrogen receptor expression could occur if receptor levels were below the sensitivity of detection but sufficient to mount an oestrogen response. The response of the majority of tumours with this profile of expression to hormonal therapy is consistent with this explanation.

The present study includes tumours that do not express oestrogen or progesterone receptors, which allows the predictive value of biomarkers to be evaluated in tumours that are oestrogen receptor-negative or that have relatively low levels of expression. Multivariate binary logistical regression analysis demonstrated that the ability of TFF3 to discriminate which patients will respond to endocrine intervention is independent of the other parameters evaluated. TFF3 is also an independent predictor of the degree of response to endocrine interventions. It remains possible that other variables were excluded from the final equation because they are highly related to TFF3. The results from the discovery cohort were validated in an independent cohort of patients with metastatic breast cancer.

There are three clinical settings in which the measurement of TFF3 could inform the clinical management of breast cancer patients. The first is for the ∼25% of patients with oestrogen receptor- and progesterone receptor-negative tumours. These patients are not eligible for endocrine therapy, although around 10% would benefit from it. Measurement of TFF3 and TFF1 would identify patients who might respond to hormonal interventions.

The second clinical setting is for patients who have oestrogen receptor levels around the threshold that defines positivity, which is currently an Allred score of 3.0. There has been debate about the most appropriate threshold and a trend to define tumours with lower oestrogen receptor content as positive (Allred 2010); a lower threshold increases the number of patients who can receive and benefit from endocrine therapy but reduces the accuracy of prediction. Knowledge of the expression of oestrogen-responsive biomarkers could assist in treatment stratification, because tumours that express TFF3, TFF1 and progesterone receptor are more likely to respond. The corollary is that absence of expression would indicate that endocrine intervention is unlikely to be effective. Such knowledge might increase patient compliance in the adjuvant setting; it is estimated that 40–50% of patients do not take their endocrine therapy (van Herk-Sukel *et al*. 2010, Hershman *et al*. 2010).

The third clinical setting is for patients with oestrogen receptor-positive tumours, of whom around 50% do not respond to endocrine therapy. The absence of progesterone receptor, TFF1 and especially TFF3 expression in patients with oestrogen receptor-positive tumours provides a strong indication that patients will not benefit from hormonal therapy. Amongst the oestrogen receptor-positive, TFF3-positive tumours, 84% responded and 68% had a partial or complete response, whereas only 37% of oestrogen receptor-positive, TFF3-negative tumours responded and none had a partial or complete response. The absence of TFF3 would provide clinicians with additional information about likely benefit from endocrine therapy. The incidence of oestrogen receptor-positive breast cancer with distant metastasis at presentation has risen recently by 2% annually in women below the age of 40 and even faster in women below the age of 35 (Johnson *et al*. 2013). Treatment of these young women with systemic, endocrine-based therapies should be informed by the best possible information obtained from analysis of combinations of biomarkers, including TFF3.

We investigated the possibility that TFF3 might be a predictive biomarker of oestrogen responsiveness because it mediates the effects of oestrogen on breast cancer metastasis. Consistent with our previous study, which showed that TFF3 is associated with lymphatic, vascular, neural and muscle invasion in breast cancer (Ahmed *et al*. 2012), TFF3 promoted cell migration and invasion. These data suggest that hormone therapy reduces tumour progression by inhibiting the synthesis of proteins, such as TFF3.

In conclusion, TFF3 expression is an independent predictive biomarker of both oestrogen response and degree of response. The evaluation of TFF3 expression in addition to oestrogen and progesterone receptors could identify additional breast cancer patients who might benefit from endocrine intervention and could inform the clinical management of patients with low oestrogen receptor positivity. Furthermore, the absence of TFF3 expression in tumours that express oestrogen receptor indicates that endocrine therapy is less likely to be effective.

## Author contribution statement

F E B May and B R Westley were jointly responsible for the conception of the study, the experimental design, the statistical analyses and the preparation of the manuscript. Both authors read and approved the final manuscript.
